# Reduced Incidence of Slowly Progressive Heymann Nephritis in Rats Immunized With a Modified Vaccination Technique

**DOI:** 10.1080/17402520600563758

**Published:** 2006-03

**Authors:** Arpad Z. Barabas, Chad D. Cole, Arpad D. Barabas, Aron N. Barabas, Rene Lafreniere

**Affiliations:** 1 Department of Surgery University of Calgary Health Sciences Centre Calgary Alberta Canada; 2 Department of Neurosurgery University of Utah Salt Lake City Utah USA

## Abstract

A slowly progressive Heymann nephritis (SPHN) was induced in three groups of rats by weekly injections of a chemically modified renal tubular antigen in an aqueous medium. A control group of rats received the chemically unmodified version of the antigen in an aqueous solution. One group of SPHN rats were pre- and post-treated with weekly injections of IC made up of rKF3 and rarKF3 IgM antibody at antigen excess (MIC) (immune complexes [ICs] containing sonicated ultracentrifuged [u/c] rat kidney fraction 3 [rKF3] antigen and IgM antibodies specific against the antigen, at slight antigen excess). One group of SPHN rats were post-treated with MIC 3 weeks after the induction of the disease and one group of SPHN animals received no treatment. The control group of rats received pre- and post-treatment with sonicated u/c rKF3.

The incidence of immune-complex glomerulonephritis (ICGN) in the untreated SPHN rats was 87%, in the pre- and post-treated animals 13%, and in the post-treated-only rats 20%. Rats receiving sonicated ultracentrifuged rKF3 antigen did not develop ICGN.

The present experiment demonstrates that the development of SPHN can be not only prevented but also effectively terminated by our newly developed modified vaccination technique.


*Clinical & Developmental Immunology*, March 2006; 13(1): 17–24

Taylor & Francis

Taylor & Francis Group

### Reduced incidence of slowly progressive Heymann nephritis in rats immunized with a modified vaccination technique

ARPAD Z. BARABAS[1], CHAD D. COLE[2], ARPAD D. BARABAS[1], ARON N. BARABAS[1], & RENE LAFRENIERE[1]

[1]*Department of Surgery, University of Calgary, Health Sciences Centre, Calgary, Alberta, Canada, and* [2]*Department of Neurosurgery, University of Utah, Salt Lake City, Utah, USA*

**Abstract**

A slowly progressive Heymann nephritis (SPHN) was induced in three groups of rats by weekly injections of a chemically modified renal tubular antigen in an aqueous medium. A control group of rats received the chemically unmodified version of the antigen in an aqueous solution. One group of SPHN rats were pre- and post-treated with weekly injections of IC made up of rKF3 and rarKF3 IgM antibody at antigen excess (MIC) (immune complexes [ICs] containing sonicated ultracentrifuged [u/c] rat kidney fraction 3 [rKF3] antigen and IgM antibodies specific against the antigen, at slight antigen excess). One group of SPHN rats were post-treated with MIC 3 weeks after the induction of the disease and one group of SPHN animals received no treatment. The control group of rats received pre- and post-treatment with sonicated u/c rKF3.

The incidence of immune-complex glomerulonephritis (ICGN) in the untreated SPHN rats was 87%, in the pre- and post-treated animals 13%, and in the post-treated-only rats 20%. Rats receiving sonicated ultracentrifuged rKF3 antigen did not develop ICGN.

The present experiment demonstrates that the development of SPHN can be not only prevented but also effectively terminated by our newly developed modified vaccination technique.

**Keywords:** *Autoimmunity, modified vaccination technique, non-pathogenic IgM autoantibody, pathogenic IgG autoantibody, slowly progressive Heymann nephritis*

**Abbreviations:** *aab, autoantibody; FCA, Freund's complete adjuvant; GBM, glomerular; FX1A, nephritogenic antigen; H&E, hematoxylin and eosin; HN, Heymann nephritis; IC, immune complex; ICGN, immune complex glomerulonephritis; IP, intraperitoneal; MIC, IC made up of rKF3 and rarKF3 IgM antibody at antigen excess; rKF3, rat kidney fraction 3; rarKF3, rat anti-rKF3; SPHN, slowly progressive Heymann nephritis; u/c, ultracentrifuged*

### Introduction

Autoimmune diseases in humans are treated mainly with immunosuppressive agents. These agents are non-specific in their action: they depress the overall function of the immune system and cause numerous side effects.

However, newer approaches have shown promise. For example, orally or nasally administering disease-related antigens has appeared in certain studies to slow down immunopathological events rather than exacerbating them (al Sabbagh et al. 1994, Yoshino et al. 1995, Weiner 1997, Hussell and Humphreys 2002). Administration of pooled immunoglobulin obtained from the sera of hundreds of normal blood donors has also seemed to have beneficial effects in patients with autoimmune disorders (Imbach et al. 1981, Becker et al. 1995, Mobini et al. 1995, Jolles 2002). Recently discovered medications that are able to eliminate autoreactive B cells and thereby suppress pathogenic autoimmune events are also promising treatment options (Saleh et al. 2000, Quartier et al. 2001).

Strictly speaking, antigen-specific down-regulation of undesirable pathogenic autoimmune responses (especially when autoimmune disease-causing processes have been ascertained) had not yet been

Correspondence: A. Z. Barabas, Department of Surgery, 2802 Health Sciences Centre, 3330 Hospital Dr NW, Calgary, Alberta, Canada T2N 4N1. Tel: 1 403 220 8901. Fax: 1 403 270 8795. E-mail: barabas@ucalgary.ca

ISSN 1740-2522 print/ISSN 1740-2530 online © 2006 Taylor & Francis
DOI: 10.1080/17402520600563758

discovered until the presently described experimental autoimmune kidney disease in rats called slowly progressive Heymann nephritis (SPHN) (Barabas et al. 2004a, Barabas and Lafreniere 2005). The goal for the last few years has been to introduce the relevant self antigen to the immune system in such a way as to provoke the desired down-regulatory effect on immune response against self. Current antigen delivery systems have not been the most suitably designed techniques to prevent and/or treat autoimmune disorders.

Recently, we have discovered and implemented a new antigen delivery system using a new vaccination methodology. We call the new technique "modified vaccination" (patent pending), since its effectiveness in evoking a desirable immune response in the injected animal is based on immune-inducing components that are employed in active and passive immunizations. We have found that immune complexes made of antigens combined with specific antibodies at slight antigen excess can produce in the vaccinated host the same class of immunoglobulin with the same antibody activity against the target antigen as occurs in the inoculum itself. This vaccination technique is able to evoke a powerful predetermined immune response in the host. We have also observed that the new vaccination protocol can be used both prophylactically and therapeutically. We believe that this modified vaccination technique holds the promise of changing or redirecting immune function for the benefit of the vaccinated host in several human medical conditions that do not yet have satisfactory treatments.

The aim of the present experiment was to implement the modified vaccination technique in a newly developed autoimmune kidney disease, called SPHN (Barabas et al. 2004b), where immunopathological events are slowly progressive, just as in some "naturally occurring" autoimmune diseases in humans (Arbuckle et al. 2003).

### Materials and methods

### *Experimental groups*

Two-month-old male Sprague Dawley rats, known to develop Heymann nephritis (HN), were used in the experiment. Prior to randomly allocating them to the four experimental groups they were earmarked for individual identification. All invasive procedures were carried out on Isoflurane anaesthetized rats. At the end of the experiment at 26 weeks, the rats were euthanized by intraperitoneal (IP) injection of Euthanyl (180 mg/kg body weight, MTC Pharmaceuticals, Cambridge, ON).

### *Control group*

Fifteen rats were injected intraperitoneally with 30 μg rKF3 antigen every week till the end of the experiment. From 3 weeks after the first injection of rat kidney fraction 3 (rKF3) they received additional weekly sonicated u/c 100 μg rKF3 antigen intraperitoneally (always 3 days post 30 μg rKF3 injections) for the first 10 weeks, and then 300 μg sonicated u/c rKF3 for the remaining weeks, in 0.2 ml saline.

### *Test group 1*

Fifteen rats were pre- and post-treated intraperitoneally with IC made up of rKF3 and rarKF3 IgM antibody at antigen excess (MIC) (containing immune complexs (ICs) made up of 30 μg rKF3 antigen and rat anti-rKF3 IgM antibody, at slight antigen excess) 3 weeks before the start of the experiment proper, and then at weekly intervals till the end of the experiment. To induce the disease, rats received weekly IP injections of 100 μg azo sonicated u/c rKF3 for the first 10 weeks, and then 300 μg azo-sonicated u/c rKF3 for the remaining weeks, in 0.2 ml saline.

### *Test group 2*

Fifteen rats were post-treated with MIC 3 weeks after the disease-inducing antigen injections, administered exactly the same way as in test group 1 rats.

### *Test group 3*

Fifteen rats received weekly injections of only the disease-causing antigens, at the same time as test group 1 and 2 rats.

### *Preparation of rKF3, sonicated u/c rKF3, azo-sonicated ultracentrifuged rKF3 and azo-sonicated rKF3 antigens*

These antigen preparations were made using Sprague Dawley rat kidney fraction 3 by methods and procedures previously described (Barabas et al. 2003).

### *Urinary protein estimation*

Twenty-four-hour specimens of urine were collected from individual rats in metabolic cages before the start of the experiment and at weeks 2, 4, 7, 8, 9, 20, 21 and 26. The protein content of the urine samples was determined by the biuret method of Weichselbaum (1946) using a Spectronic Genesis 5 Spectophotometer at 540 nm.

### *Light microscopy, direct immunofluorescence and electronmicroscopy on renal cortical samples*

Kidney biopsies were obtained 8 weeks after the induction of SPHN from 15 rats pre- and post-treated with MIC, and from 5 rats post-treated only. Biopsies were also obtained from 15 untreated SPHN rats and from 4 rats injected only with rKF3. Kidney sections were stained by the direct fluorescence antibody tests for rat IgG employing appropriate dilutions of Alexa Fluor 488-labeled goat anti-rat IgG (H + L) (Molecular Probes Inc., Eugene, OR). At the end of the experiment, histological specimens were obtained for H&E and methenamine silver staining from kidney samples of 9 of each 15 test group rats and 5 of the 15 control group rats. Representative kidney biopsy samples were also processed for electron microscopy.

### *Preparation of rat anti-rat KF3 IgM and immune complexes (designated as MIC)*

Production of rat anti-rat KF3 IgM antibody was carried out in rats by repeated IP injections of rKF3 antigen in an aqueous medium. Immune complexes, designated as MIC (made up of rKF3 antigen and rarKF3 IgM antibody at slight antigen excess), were prepared prior to the injections of test group 1 and 2 rats as previously described (Barabas et al. 2004a, Barabas and Lafreniere 2005).

### *Grading of glomerular lesions resulting from deposition of rat IgG*

The intensity of fluorescence in the glomeruli was determined on a scale of 0–4 + by a semi-quantitative method at a constant microscope setting. The amount of fluorescent material in the glomeruli was also graded. Grade 0 lesions had no glomerular deposits. Most of the ICs in the glomerular capillaries of rats with immune complex glomerulonephritis (ICGN) were made up of small closely packed deposits. The differences in the amounts of single-layered deposits were quantified on a 0–4 + scale. The single layer of deposit around the glomerular capillaries indicated that the lesions were not too advanced.

### Results

### *Proteinuria*

One pre-treatment sample of urine was obtained from each group of rats to find out if pre-treatment with rKF3 or MIC would effect protein excretion. The average proteinuria levels of the four groups of rats, as ascertained from the pre-SPHN induction urine samples, was between 5 and 6 mg per 24 h. Results obtained at the end of the experiment from individual groups of rats similarly showed no significant change in protein excretion levels, being between 7 and 10 mg per 24 h on average. Thus, neither pre-/post- or post-treatment with MIC nor injection of azo-rKF3 or rKF3 antigens significantly altered protein excretion during the 26-week experimental period.

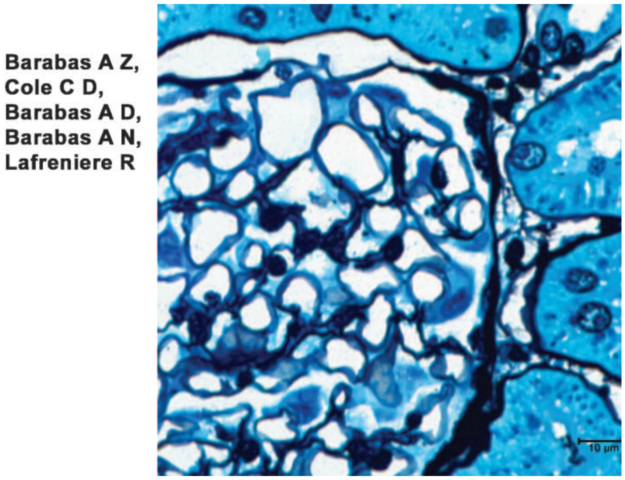

Figure 1. Evenly thin glomerular capillary loops show no morphological changes in the kidney sections of test and control group rats. PASM stained section.

### *Light microscopy*

Hematoxylin and eosin Hematoxylin and eosin (H&E) kidney sections of variously treated rats showed no differences in glomerular cell counts. On average these ranged from 48 to 51 cells per glomerulus in each of the four experimental groups of rats. Methenamine silver-stained kidney sections similarly revealed no detectable changes in the glomerular capillary blood vessels (Figure 1), except in the two rats with the highest grade lesions. These rats, and one of them in particular, showed numerous vaccuolations in their glomerular capillary loops, revealing the early changes typically observed in HN kidney lesions.

### *Direct fluorescence antibody test results*

Rat kidney biopsies obtained 8 weeks after the induction of SPHN showed the following: in the untreated group of rats all except one rat (14 of 15) had detectable beaded deposits staining for rat IgG, mainly with faint fluorescence, along the glomerular capillary loops. The lesions were considered to be of low grade. In the pre-/post-treated rats, 12 out of 15 had no detectable deposits in the glomeruli. The three kidney specimens which did stain showed small, faint, sparsely distributed deposits. Only five biopsies were obtained (of 15 animals) from the post-treated group of rats. Three kidney specimens showed glomerular deposits that were mostly faint and linear but in some areas small and beaded, and two samples were completely negative. None of the four kidney samples obtained from the rKF3 injected rats had any glomerular deposits.

At the end of the experiment kidney samples from each rat were also stained for the presence of rat IgG. The incidence of SPHN and fluorescence-graded

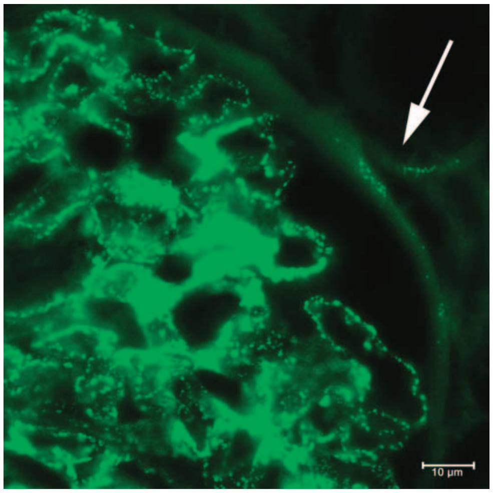

Figure 2. Kidney section of an SPHN untreated rat stains for rat IgG in the glomerular capillary loops with a diffuse beaded pattern of fluorescence by a direct fluorescence antibody test. The Bowman's capsule and the tubular basement membrane also stain with beaded deposits (white arrow).

glomerular lesions are shown on the table. It can be noted that 13 of 15 rats in the SPHN untreated rats had detectable glomerular deposits (Figure 2), while in the pre-/post- and post-treated-only rats only 2 of 15 and 3 of 15, respectively, had beaded deposits (Figure 3—Left). Rats injected with rKF3 antigen had no deposits in the glomeruli (Figure 3—Right). The table presenting the grade scores clearly shows that both pre-/post-treatment and post-treatment only greatly prevented the occurrence of glomerular deposits.

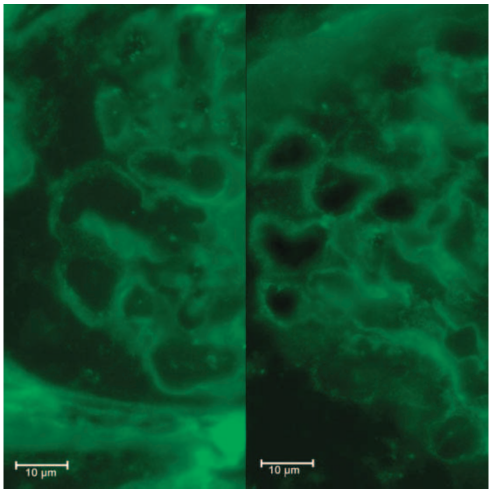

Figure 3. Kidney sections of pre-/post-induction and post-induction-only treated SPHN rats (left) and of rKF3 antigen injected rats (right) show no depositions of rat IgG in the glomerular capillaries by direct fluorescence antibody testing (overexposed pictures).

*Electron microscopy*

Ultrathin rat kidney sections with no beaded depositions of rat IgG in the glomeruli by fluorescent antibody tests also showed no ultrastructural changes (Figures 4 and 5). Animals with low fluorescence grades for rat IgG presence in the glomeruli had detectable small deposits on the epithelial side of the glomerular basement membrane. As the fluorescence grades increased, the size of the deposits also increased. The most severe glomerular lesions were observed in the kidneys of untreated SPHN rats (Figure 6). In these animals, small to medium-sized deposits were observed on the epithelial side of the glomerular basement membrane (GBM). Epithelial cell foot processes showed fusion in relation to the larger deposits. A few of the pre-/post-treated and post-treated-only rat kidneys had mild glomerular lesions but most of them, just like the rKF3 antigen injected rats (Figure 7), had no detectable ultra-structural kidney alterations.

### Discussion

HN is an experimental autoimmune kidney disease of rats. It is initiated and maintained by the development of pathogenic IgG autoantibodies (aabs) following IP injections of renal tubular antigenic preparations (FX1A, rKF3, etc.) incorporated mainly in Freund's complete adjuvant (FCA) (Heymann et al. 1959, Grupe and Kaplan 1969, Barabas et al. 2003). A similar more slowly progressive HN called SPHN can also be established by two different methods described by Barabas and associates (Barabas et al. 2003, 2004b). SPHN is also induced by the development of pathogenic aabs, but because the nephritogenic antigen is introduced in Alum or in a chemically modified format, the initiation and progression of the disease is considerably slower (Barabas et al. 2003, 2004b). In many ways, especially when the chemically modified nephritogenic antigen is administered in an aqueous medium, the slow development of the autoimmune disease resembles spontaneously occurring auto-immune-inducing events in humans (Arbuckle et al. 2003). The slow development of SPHN allows sufficient time to better study the immunopathological events which contribute to disease progression (Barabas et al. 2003). Moreover, there is a better chance in SPHN to intervene and downregulate or even terminate auto-immune disease-causing events (Barabas et al. 2004a, Barabas and Lafreniere 2005). Previous attempts to treat HN with immunosuppressive agents either before or after its induction resulted in no significant changes in the three most important aspects of the disease (Barabas et al. 1970, Kupor et al. 1976, Cattran 1988, Matsukawa et al. 1992, Yokoyama et al. 1999). In these experiments reductions in proteinuria, decreases in circulating pathogenic IgG aab levels and morpho-

Barabas A Z, Cole C D, Barabas A D, Barabas A N, Lafreniere R

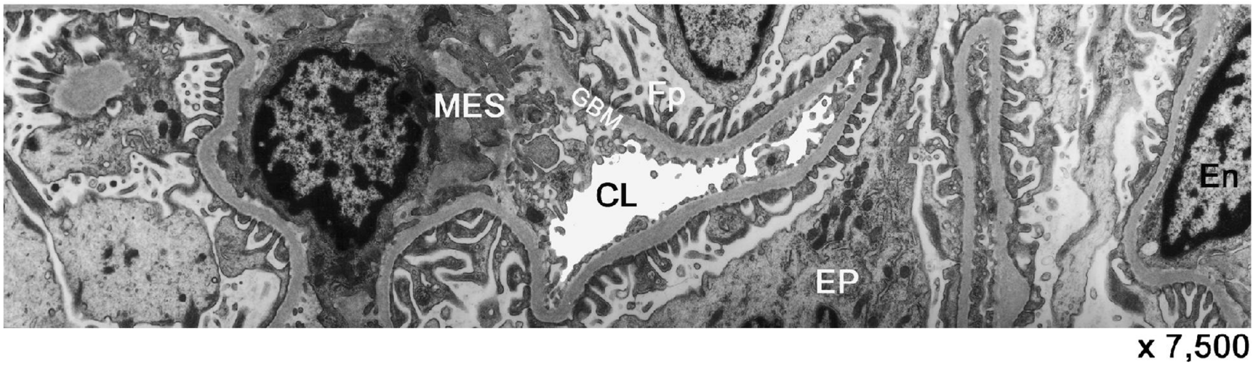

Figure 4. Glomerular capillary loops of an SPHN pre-/post-treated rat showing evenly thin GBMs, preserved foot processes, and no change in the epithelial cells ( × 7500). CL, capillary-loop; D, deposit; En, endothelial cell; Ep, epithelial cell; Fp, foot process; GBM, glomerular basement membrane; MES, mesangial cell.

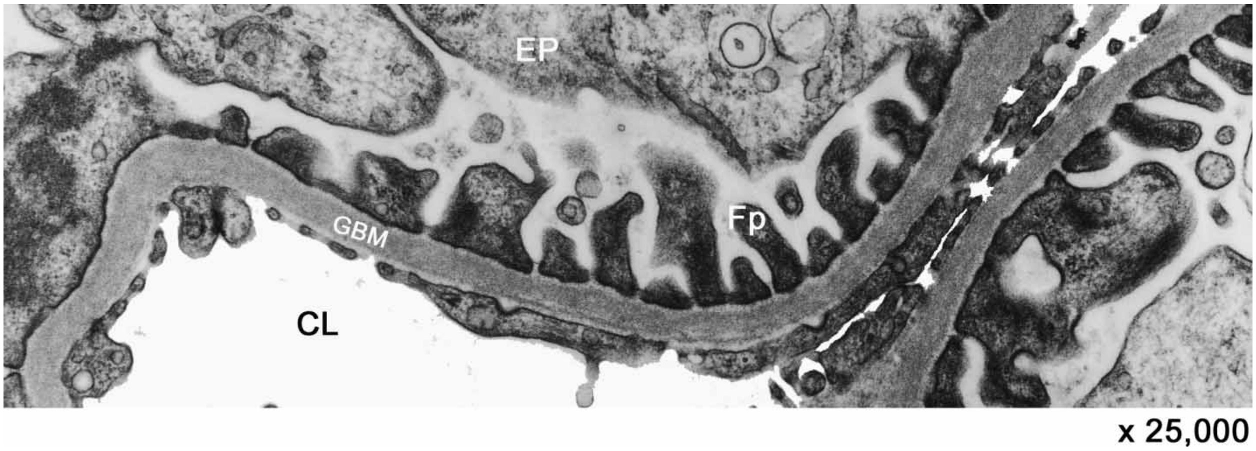

Figure 5. Glomerular capillary loop of an SPHN post-treated-only rat. Evenly thin GBM, well preserved foot processes, and epithelial cells opposite the GBM show no morphological changes ( × 25,000). CL, capillary-loop; Ep, epithelial cell; Fp, foot process; GBM, glomerular basement membrane.

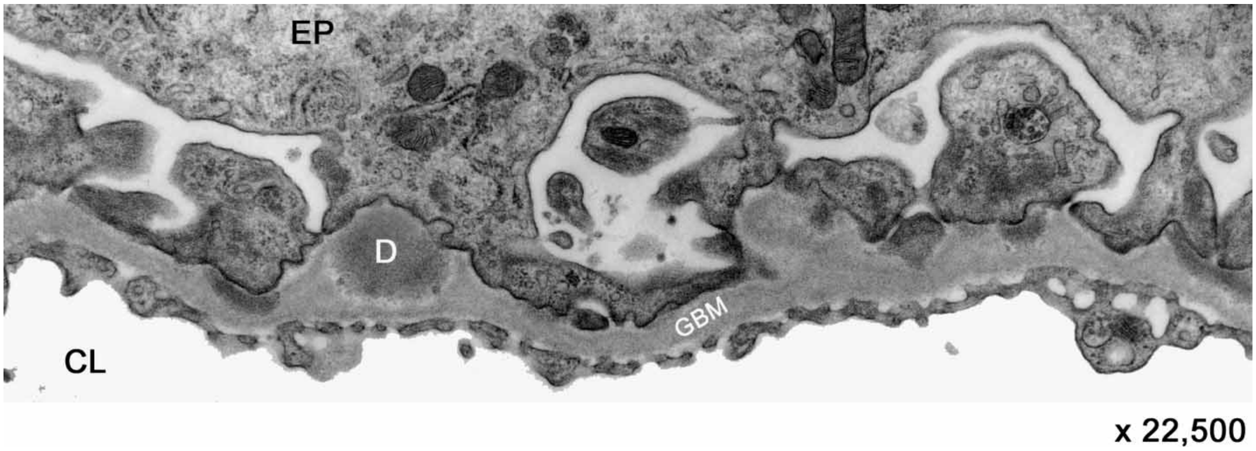

Figure 6. Glomerular capillary loop of an SPHN untreated rat. Small to large osmiophilic deposits are partially surrounded by BM-like material on the epithelial side of the minimally thickened GBM. Foot processes are fused opposite the deposits and the epithelial cell cytoplasm over the deposits shows mild signs of osmiophilia ( × 22,500). BM, basement membrane; CL, capillary-loop; D, deposit; Ep, epithelial cell; GBM, glomerular basement membrane.

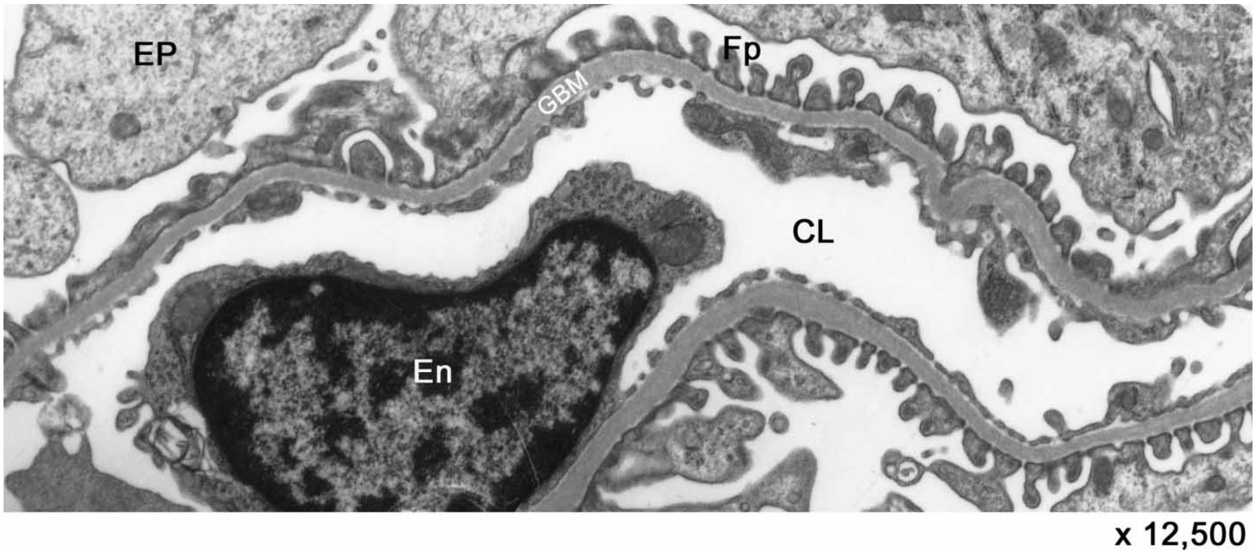

Figure 7. Glomerular capillary loops of a control rKF3 antigen-injected rat. The glomerular capillary loop and its related structures show no morphological alterations ( × 12,500). CL, capillary-loop; En, endothelial cell; Ep, epithelial cell; Fp, foot process; GBM, glomerular basement membrane.

logical changes in the kidney did not occur. There have been several attempts lately to intervene more specifically with immune events in order to suppress disease-causing processes. To our knowledge none of these techniques have so far been specific enough to cause substantial reductions in disease progression without significant side effects.

Our modified vaccination technique, on the other hand, is expected to have a major impact, far beyond the downregulation of our experimental autoimmune kidney disease, on the treatment of many human autoimmune diseases that are mediated by pathogenic aabs as well. Utilizing the observations of Grabar (1957, 1965) and the very extensive work of Weir and associates (Pinckard and Weir 1966, Weir 1966, Weir et al. 1966, Elson and Weir 1967, Weir and Pinckard 1967, Weir and Elson 1969), wherein, it was noted that released intracytoplazmic components are assisted in their removal from the circulation by specific IgM aabs, we set out to find out if a specific increase in IgM aab production against nephritogenic autoantigens was able to remove or block circulating modified and unmodified nephritogenic antigens. A specific increase in IgM aab production might, by removing nephritogenic antigens from the circulation, even terminate pathogenic IgG aab production.

We have recently shown that immuno-pathological events can be down-regulated by injections of MIC (containing the native nephritogenic antigen and specific IgM antibody against it at a slight antigen excess) in SPHN rats (Barabas et al. 2004a, Barabas and Lafreniere 2005). We maintain that the down-regulatory effect of MIC is due to its ability to specifically up-regulate IgM aab production against the nephritogenic aags (modified or unmodified). Increased production of IgM aabs results in an unusually high level of circulating IgM aabs, which are able to assist in the removal of nephritogenic antigens and thereby prevent further production of pathogenic disease-causing IgG aabs.

In the present experiment, we investigated whether the injected MIC could influence the course of a milder form of SPHN (Barabas et al. 2004b) in pre-/post-treated and post-treated-only rats. SPHN untreated and rKF3 antigen injected rats were also included in the experiment. During the 26-week experimental period none of the treated or untreated SPHN rats or the control rats developed proteinurias. The low proteinuria results suggested that the glomerular lesions observed by histology, direct fluorescence antibody tests and electron microscopy would not be too severe, and our findings accorded with this prediction.

Experimental results at the end of the experiment at 26 weeks clearly revealed that pre-/post-induction treatment and post-induction-only treatment with MIC can substantially reduce the incidence and severity of the developing autoimmune disease in rats, to 13 and 20% ICGN occurrence in our test groups, respectively, compared to an 87% incidence in our untreated rats (Table I). This experiment also proved once more that the continual injection of unmodified renal tubular antigen rKF3 will not cause disease (Barabas et al. 2004b). It had also been shown earlier that the continuous release of “unmodified” intracytoplazmic components following cell death will maintain only non-pathogenic IgM aab production and not pathogenic IgG aab production in normal physiological conditions (Weir et al. 1966, Weir and Pinckard 1967).

Table I. Incidence of SPHN and grade of glomerular IgG antibody deposition in SPHN treated and untreated rats and in control rats at the end of the experiment at 26 weeks.

| Groups of rats and treatments | Number of rats in group | Number of rats w/ICGN | Incidence of SPHN(%) | Number of rats with glomerular lesion grades | | | | | |
|---|---|---|---|---|---|---|---|---|---|
| | | | | <0.5 | 0.5–1 | 1–1.5 | 1.5–2 | 2–2.5 | 2.5–3.5 |
| *Test groups* | | | | | | | | | |
| I SPHN p/p MIC Tx | 15 | 2 | 13 | 2 | – | – | – | – | – |
| II SPHN p MIC Tx | 15 | 3 | 20 | 1 | – | – | 1 | – | 1 |
| III SPHN unTx | 15 | 13 | 87 | 3 | 3 | 1 | 1 | 3 | 2 |
| *Control group* | | | | | | | | | |
| Rats inj. w/rKF3 | 15 | 0 | 0 | – | – | – | – | – | – |

In this experiment lesion grades of 0–4 represent the number and size of deposits around the glomerular capillaries in direct fluorescence antibody testing for rat IgG; 0.5: few small faintly staining deposits around the glomeruli; 0.5–2: still few but more frequently displayed single layered small deposits around the glomeruli; 2–4: small to large closely packed single-layered deposits around the glomeruli; p: post-; p/p: pre- and post-; Tx: treated; unTx: untreated.

So far no treatment options of any kind had been able to terminate the immunopathological events that led towards full-blown autoimmune diseases characterized by structural and functional changes in the target organ. Our unique vaccination technique therefore represents a significant breakthrough. As we have also shown in previous experiments (Barabas et al. 2004a, Barabas and Lafreniere 2005), this technique is able, by the injection of ICs called MICs (which contain the relevant immune response-inducing components), to initiate in the injected host the production of the same class of antibody with the same specificity against the antigen that resides in the injected IC itself. The subsequent development of IgM aabs specific against the target antigen assists in the catabolism of both the modified (injected) and unmodified (released from renal tubules) antigens present in the circulation. By reducing and in most cases completely eliminating the modified antigen from the circulation, the production of tissue-damaging pathogenic IgG aabs is prevented. We believe that our experiments represent the first time that a treatment modality has been able to specifically affect the autoimmune disease-causing events and thereby result in the prevention and/or termination of an experimental autoimmune kidney disease. Unlike presently available treatments, this new vaccination technique, when its IC components are properly assembled and employed, will not interfere with the overall function of the immune system, and nor will it cause side effects.

This modified vaccination technique, as we have observed in our present and previous experiments, holds the promise of providing a chance for vaccinating both prophylactically and therapeutically against both exogenous and endogenous source derived antigens. Our newly implemented modified vaccination technique could in the very near future provide better treatment options for curing autoimmune disorders, chronic infections and even cancer.

### Acknowledgements

We acknowledge the very competent assistance of Twyla Boehmer and of Mr Z. Kovacs in technical and computer-related work.

### References al Sabbagh A, Miller A, Santos LM, Weiner HL. 1994. Antigen-driven tissue-specific suppression following oral tolerance: Orally administered myelin basic protein suppresses proteolipid protein-induced experimental autoimmune encephalomyelitis in the SJL mouse. Eur J Immunol 24: 2104–2109.

Arbuckle MR, McClain MT, Rubertone MV, Scofield RH, Dennis GJ, James JA, Harley JB. 2003. Development of autoantibodies before the clinical onset of systemic lupus erythematosus. N Engl J Med 349:1526–1533.

Barabas AZ, Cole CD, Barabas AD, Lafreniere R. 2003. Production of a new model of slowly progressive Heymann nephritis. Int J Exp Pathol 84:245–258.

Barabas AZ, Cole CD, Barabas AD, Lafreniere R. 2004a. Down-regulation of pathogenic autoantibody response in a slowly progressive Heymann nephritis kidney disease model. Int J Exp Pathol 85:321–334.

Barabas AZ, Cole CD, Barabas AD, Lafreniere R. 2004b. Production of Heymann nephritis by a chemically modified renal antigen. Int J Exp Pathol 85:277–285.

Barabas AZ, Lafreniere R. 2005. Antigen-specific down-regulation of immunopathological events in an experimental autoimmune kidney disease. Autoimmun Rev 4:565–570.

Barabas AZ, Nagi AH, Lannigan R, Womersley RA. 1970. The effect of cortisone treatment on autologous immune complex glomerulonephritis in rats. Br J Exp Pathol 51: 541–546.

Becker BN, Fuchs H, Hakim R. 1995. Intravenous immune globulin in the treatment of patients with systemic lupus erythematosus and end-stage renal disease. J Am Soc Nephrol 5:1746–1750.

Cattran DC. 1988. Effect of ciclosporin on active Heymann nephritis. Nephron 48:142–148.

Elson CJ, Weir DM. 1967. Chemotaxis of polymorphs induced by tissue antigens and normal serum in rats: A possible clearance mechanism. Clin Exp Immunol 2:581–588.

Grabar P. 1957. The problem of auto-antibodies; An approach to a theory. Tex Rep Biol Med 15:1–16.

Grabar P. 1965. Some considerations of the problem of auto-antibody formation. Tex Rep Biol Med 23:278–284.

Grupe WE, Kaplan MH. 1969. Demonstration of an antibody to proximal tubular antigen in the pathogenesis of experimental autoimmune nephrosis in rats. J Lab Clin Med 74:400–409.

Heymann W, Hackel DB, Harwood S, Wilson SG, Hunter JLP. 1959. Production of the nephritic syndrome in rat by Freund's adjuvant and rat kidney suspension. Proc Soc Exp Biol Med 100:660–664.

Hussell T, Humphreys IR. 2002. Nasal vaccination induces protective immunity without immunopathology. Clin Exp Immunol 130:359–362.

Imbach P, Barandun S, d'Apuzzo V, Baumgartner C, Hirt A, Morell A, Rossi E, Schoni M, Vest M, Wagner HP. 1981. High-dose intravenous gammaglobulin for idiopathic thrombocytopenic purpura in childhood. Lancet 1:1228–1231.

Jolles S. 2002. High-dose intravenous immunoglobulin (hdIVIg) in the treatment of autoimmune blistering disorders. Clin Exp Immunol 129:385–389.

Kupor LR, Lowance DC, McPhaul JJ. Jr, 1976. Single and multiple drug therapy in autologous immune complex nephritis in rats. J Lab Clin Med 87:27–36.

Matsukawa W, Hara S, Yoshida F, Suzuki N, Fukatsu A, Yuzawa Y, Sakamoto N, Matsuo S. 1992. Effects of a new immunosuppressive agent, FK506, in rats with active Heymann nephritis. J Lab Clin Med 119:116–123.

Mobini N, Sarela A, Ahmed AR. 1995. Intravenous immunoglobulins in the therapy of autoimmune and systemic inflammatory disorders. Ann Allergy Asthma Immunol 74:119–128.

Pinckard RN, Weir DM. 1966. Antibodies against the mitochondrial fraction of liver after toxic liver damage in rats. Clin Exp Immunol 1:33–43.

Quartier P, Brethon B, Philippet P, Landman-Parker J, Le Deist F, Fischer A. 2001. Treatment of childhood autoimmune haemolytic anaemia with rituximab. Lancet 358:1511–1513.

Saleh MN, Gutheil J, Moore M, Bunch PW, Butler J, Kunkel L, Grillo-Lopez AJ, LoBuglio AF. 2000. A pilot study of the anti-CD20 monoclonal antibody rituximab in patients with refractory immune thrombocytopenia. Semin Oncol 27:99–103.

Weichelbaum TD. 1946. Accurate and rapid method for determination of proteins in small amounts of blood, serum and plasma. Am J Clin Path Tech Suppl 10:40–49.

Weiner HL. 1997. Oral tolerance for the treatment of autoimmune diseases. Annu Rev Med 48:341–351.

Weir DM. 1966. The immune response after tissue injury. Pathol Eur 1:108–118.

Weir DM, Elson CJ. 1969. Antitissue antibodies and immunological tolerance to self. Arthritis Rheum 12:254–260.

Weir DM, Pinckard RN. 1967. Failure to induce tolerance to rat tissue antigens. Immunology 13:373–380.

Weir DM, Pinckard RN, Elson CJ, Suckling DE. 1966. Naturally occurring anti-tissue antibodies in rat sera. Clin Exp Immunol 1:433–442.

Yokoyama H, Goshima S, Wada T, Takaeda M, Furuichi K, Kobayashi K, Kida H. 1999. The short- and long-term outcomes of membranous nephropathy treated with intravenous immune globulin therapy. Kanazawa study group for renal diseases and hypertension. Nephrol Dial Transplant 14:2379–2386.

Yoshino S, Quattrocchi E, Weiner HL. 1995. Suppression of antigen-induced arthritis in Lewis rats by oral administration of type II collagen. Arthritis Rheum 38:1092–1096.

